# In Bilinguals' Hands: Identification of Bilingual, Preverbal Infants at Risk for Language Delay

**DOI:** 10.3389/fped.2022.878163

**Published:** 2022-06-02

**Authors:** Carina Lüke, Ute Ritterfeld, Ulf Liszkowski

**Affiliations:** ^1^Special Education and Therapy in Language and Communication Disorders, Faculty of Human Sciences, University of Würzburg, Würzburg, Germany; ^2^Department of Language and Communication, Faculty of Rehabilitation Sciences, TU Dortmund University, Dortmund, Germany; ^3^Department of Developmental Psychology, Institute of Psychology, University of Hamburg, Hamburg, Germany

**Keywords:** gesture, pointing, language delay, language acquisition, bilingualism

## Abstract

Studies with monolingual infants show that the gestural behavior of 1–2-year-olds is a strong predictor for later language competencies and, more specifically, that the absence of index-finger pointing at 12 months seems to be a valid indicator for risk of language delay (LD). In this study a lack of index-finger pointing at 12 months was utilized as diagnostic criterion to identity infants with a high risk for LD at 24 months in a sample of 42 infants growing up bilingually. Results confirm earlier findings from monolinguals showing that 12-month-olds who point with the extended index finger have an advanced language status at 24 months and are less likely language delayed than infants who only point with the whole hand and do not produce index-finger points at 12 months.

## Introduction

Gestures are one of the most important precursors of linguistic skills in young children. Toward the end of the first year of life infants use gestures to communicate intentionally with others. They initiate situations of joint attention and direct the attention of their caregivers to something they are interested in or want to communicate about. Colonnesi et al. (1) showed with their meta-analysis that especially the early use of pointing gestures is predictive for later language skills. Infants who produced between 10 and 20 months a high rate of pointing gestures had better linguistic skills between 12 and 54 months compared to infants who did not use as many pointing gestures at this early age.

Further research confirmed the predictive value of pointing gestures for later language skills (2–5). For example, Kuhn et al. (2) revealed with an epidemiological sample of over 1,000 infants studied within a prospective longitudinal study, that individual differences in communicative gestures at 15 months predict language skills at 2 and 3 years of age. Similarly, a recent meta-analysis by Kirk et al. (6) found a predictive function of early pointing for later language abilities, but with a considerably smaller effect size compared to Colonnesi et al.'s meta-analysis (1). Moreover, differences in the gestures between children with typical language development and children with language delay (LD) were identified (5, 7–10). About 20% of 2 year old children have been reported to have significant delays in their language acquisition (11). Especially children whose LD manifests in a developmental language disorder (DLD), which is the case for about 40% of the children (12), face negative and long-term effects on academic achievement or mental health, and consequently, in social participation (13).

Early pointing gestures in infants take on distinct hand shapes. Liszkowski and Tomasello (14) found in a sample of monolingual 12-month-olds that the canonical shape of index-finger pointing, as opposed to whole-hand pointing, revealed advanced prelinguistic communicative competencies. Infants who pointed with the extended index finger had a better understanding of communicative intentions of their communication partner, pointed more frequently and accompanied their pointing more often with vocalizations than infants who only pointed with the whole hand. Early pointing behavior, especially in the later emerging canonical form of index-finger pointing, is a milestone in social-cognitive development [for an overview see (15)] and is predicted by infants' prior social-cognitive ability to follow the gaze direction of a communication partner (16).

Subsequent studies explored the prediction of these distinct pointing shapes to language competence. Lüke et al. found in a monolingual sample that infants at 12 months, who only pointed with the whole-hand shape but not yet canonically with the extended index finger, were more likely to have a language delay (LD) at 24 months and to have lower language skills up to the age of 6 years compared to infants who produced index-finger points at the early age of 12 months (5, 10, 17). In two high risk populations—siblings of children with an Autism Spectrum Disorder diagnosis and preterm born infants with an extremely low gestational age—Sansavini et al. (9) established that those children who turned out to have a LD between 24 and 36 months produced fewer index-finger points at 18 months of age than typical developing (TD) children. The predictive value of index-finger pointing for later language skills in children with Autism Spectrum Disorder was also found by Ramos-Cabo et al. (18).

The absence of index-finger points at a certain age (i.e., 12 months), the age of onset of index-finger pointing (19), and the number of pointing gestures at a certain age might be valid criteria for an early and cost-effective identification of infants with a high risk of LD. The usage of these criteria within a screening would be of high clinical importance since children with LD develop lower language and literacy skills than TD children throughout school years and into adolescence [e.g., (20, 21)].

Many studies indicate that parent guided intervention programs are effective to support language acquisition in children with diagnosed LD [for a meta-analysis see (22)]. We argue that such secondary preventive intervention trainings could also be facilitative for even younger children with a high risk for LD within the second year of life. To provide such secondary preventive interventions, a screening tool for identifying infants with a high risk for LD is needed. Especially in bi- and multilingual children such a screening tool would be of tremendous value as the identification of a LD or a DLD is particularly challenging given the heterogeneity of the developmental pathways of bi- and multilingual children (23, 24). Building on the data on gesture development, we argue that preverbal communication such as early gestures could be an innovative, language independent indicator of LD, also valid in bilingual children.

However, in order to establish this argument, it needs to be proven that infant pointing is similar across monolingual and bilingual samples in terms of frequency, use, and shape. So far, Liszkowski et al. (25) demonstrated that prelinguistic gesturing can be considered a universal part of communication: 10–14-month-old infants from seven very different cultures and languages (from Papua New Guinea, Indonesia, Japan, Peru, Mexico (Tzeltal and Yucatec), and Canada) used in a standardized setting pointing gestures with the same canonical form, i.e., index-finger points, and with a similar frequency, and it correlated with their caregivers' pointing for them. While Tamis-LeMonda et al. (26) found differences in the number of gestures produced by Mexican American, Dominican American and African American mothers in interaction with their 14- and 24-months-old children, no differences were found between the number of gestures produced by the children from these three different cultural backgrounds. Germain et al. (27) verified this finding and found no differences in gestural development in French and English mono- and bilinguals living in Canada, assessed with different versions of the MacArthur-Bates Communicative Development Inventories CDI (28). Cameron-Faulkner et al. (29) compared gestural behavior in 10–12-months-olds and their mothers from three culturally distinct groups (Bengali, Chinese, and English) in the United Kingdom. They also found no differences in infants' gestural development, including index-finger pointing between these groups, and a positive correlation between gestures productions at 10–12 months and lexical skills at 18 months of age. Salomo and Liszkowski (30) on the other hand, analyzed index-finger pointing during different, natural daily activities in 8–16 months old Yucatec Mayan, Dutch, and Chinese infants and found differences in the frequency of index-finger pointers. While only 27% of the Mayan infants produced index-finger points, 72% of the Dutch and 88% of the Chinese infants did. In their recent meta-analysis, Kirk et al. (6) criticized that “none of the studies included samples of bilingual infants” (p. 2). Apart from the recently published study by Cameron-Faulkner et al. (29) no data on pointing behavior in bilingual infants and its predictive value for later language competencies is currently available.

If non-verbal communication ought to be used for screening in bilinguals the parallelism of gesture communication within both language situations must be assured. A study with a sample of five French-English bilingual boys between 2.0 and 3.6 years of age suggested that the frequency of gesture-speech productions was similar in the two languages (31). In a recent study, Limia et al. (32) found comparable results in 34 Spanish-English bilingual children within nearly the same age range (2.6–3.6 years). Moreover, the authors extended the meaningfulness of their insights from this bilingual sample by comparing them with 34 monolingual children growing up with either Spanish or English: The bilingual children referred uniquely gesturally to items in their surrounding with a similar proportion as the monolingual children. Also, their proportion of unique gestures was comparable in both languages even if one language was stronger. However, insights about the very early use of pointing gestures—around 12 months of age—and their predictive value for later language competencies are still missing for bilingual children [c.f. (6)].

In the current study we took a first step toward closing this gap and collected gestural behavior of 12-month-old infants growing up bilingually and assessed their language competencies a year later, at 24 months of age. We employed the standardized paradigm from Liszkowski et al. (25) to see whether the findings on pointing of monolingual infants pertain to bilingual infants; and to assess whether the relation between index-finger pointing and language delay found by Lüke et al. (5) in monolingual infants would also pertain to a bilingual sample. Specifically, we expect higher language skills at 24 months in children who produced index-finger points 1 year before and a lower rate of children with LD in this group of children compared to children who did not use index-finger points at 12 months. If infants growing up bilingually would show a similar developmental pattern as monolingual infants, this would enable practitioners in applied settings to use similar criteria to predictively assess mono- and bi-lingual infants' language development. It would also suggest that at this initial stage of development, language exerts less influence and instead rather “piggy-backs” on its gestural and social-cognitive infrastructure (33).

## Methods

### Participants

Fifty-one 12-months-olds and their primary caregivers (90% mothers) were recruited for this longitudinal study. Data of nine children had to be excluded from analyses because they did not participate in both test sessions. The final sample included 42 infants (25 girls, 17 boys). At the first measurement, mean age of infants was 12 months and 9 days (*SD* = 10 days) and at the second measurement, mean age was 24 months and 17 days (*SD* = 14 days). All children were living together with their mothers and fathers and raised as bi- or multilingual speakers since birth. Using the graphical parent questionnaire *Input Contexts in Multilingualism* [ICOM; (34)] the language use of mothers and fathers in interaction with their infant was measured in detail. All children had exposure to German and at least to one other language on regular basis since birth. The majority of the children (76%) grew up with two languages, while 24% of the children grew up with three or more languages. Besides German, twenty different languages were spoken by the children's families with Turkish (29%), Spanish (14%), English (9.5%), and Chinese (7%) as the most prevalent languages. Albanian, Amharic, Arabic, Bulgarian, Dutch, French, Hindi, Italian, Korean, Macedonian, Pashto, Persian, Romanian, Russian, Urdu, and Vietnamese were spoken in one or two families each.

According to the pediatricians of the infants and a standardized test of global development (*Entwicklungstest für Kinder von 6 Monaten bis 6 Jahren -ET 6-6*) (Developmental test for children between 6 months and 6 years) (35) all children were typically developing. They grew up in families with a rather high level of education (*M*_maternalyearsofeducation_ = 16, *SD*_maternalyearsofeducation_ = 2; *M*_paternalyearsofeducation_ = 16, *SD*_paternalyearsof‘education_ = 3) and a household disposable income per month (*Md* = € 1.944, *IQR* = € 1.865) comparable to the German median in the same year (36).

### Procedure

#### Eliciting and Coding of Pointing Gestures

Infants and their primary caregiver took part in two sessions of data collection, one at the age of 12 months, one at 24 months. At 12 months the pointing behavior of the children was captured, using the same setting as in 5 (5), the so called “decorated room” (14). In this room 19 interesting objects and pictures are placed to elicit natural, multimodal interaction between caregivers and their infants. Caregivers were asked to carry their child for 6 min and to look at the items without touching them, if possible. Caregivers were not informed that gestures were analyzed. Four cameras recorded the scene from the four corners of the room.

A research assistant unaware of the research question coded the videos in both real-time and frame-by-frame analyses for the occurrence of pointing gestures using the annotation tool ELAN (37). Pointing was coded when the infant extended the hand and the arm toward an object or a picture without grabbing or touching it. The gestures were coded either as index-finger points—when the index finger was clearly extended relative to all other fingers—or as hand points—when the index finger was not clearly extended relative to the other fingers. To assess inter-rater reliability a second research assistant coded independently a random 10% of the sample data. Inter-rater reliability for infants' pointing was very good (Krippendorff's α = 0.967).

Based on the reliable coding of the hand shape, infants were classified as *index-finger pointers* if they pointed at least once with the index finger. Infants who only pointed with their whole hand were classified as *hand pointers* (14). Validity for group assignment was assured by an additional procedure: In all cases where infants only pointed with the whole hand or pointed just once or twice with the index finger (20 cases), the video was coded by the second independent coder. Comparisons revealed that the two codings did overlap in all but one case. In this case of non-agreement, the vote of an additional independent third coder decided upon the final group assignment. All coders were uninformed about the research question and the language status of the infants.

#### Language Testing and Diagnosis of LD

Verbal skills of the children were assessed at 24 months using standardized German language measures: *Sprachentwicklungstest für zweijährige Kinder (SETK-2)* [test of language acquisition for 2-year-old children] (38) and the *Fragebogen zur frühkindlichen Sprachentwicklung (FRAKIS)* [German equivalent of the standardized parent questionnaire CDI (28)] (39). The SETK-2 (38) consists of four subtests assessing comprehension and production of words and sentences. Results are presented in standard T-scores. Since the children in this study were raised as bilingual speakers some adaptions within the language testing were made. For the three subtests *word comprehension, sentence comprehension*, and *word production* the child got the instruction in German as well as in their other language. The test items were first presented by the experimenter in German. If the child did not react or gave a wrong answer, the caregiver provided the item again in the other language spoken within the family. This procedure was feasible because the presented items were basic words or sentences which could be easily translated by the caregivers. Beyond that, the majority of the presented words have a comparable age of acquisition in many different languages [c.f. (40)]. The procedure was explained to the caregivers before testing. The procedure of the fourth subtest *sentence production* was too complex to be translated and presented by the caregivers so that this subtest was only administered in German with those children who were able to comply (*n* = 24).

From the German parental questionnaire FRAKIS (39) the vocabulary checklist of 600 words was used. Parents were asked to indicate which of the presented 600 words were spoken by their child in either of the child's active languages. The parents marked the spoken words in the different languages by using different colored pencils, one for each language. For analyses the conceptual vocabulary as a composite of all items spoken in at least one language (41) was calculated for each child. This procedure was used since there were not for all languages in our sample adaptions of the CDI (28) available, all parents in our sample had so much German proficiency that they could answer the questionnaire easily in German and to avoid an influence by different word lists.

Paralleling the procedure with a monolingual sample (5), we defined a 2-year-old child as language delayed if s/he scored in at least one of the three standardized language subtests (*word comprehension, sentence comprehension, word production*) of the SETK-2 (38) or in the vocabulary checklist 1½ standard deviation below the mean (i.e., T-score of ≤ 35) and in at least one additional subtest or the vocabulary checklist 1 standard deviation below the mean (i.e., *T*-score of <40). By this definition 24% of the children in the sample were classified as language delayed at 24 months. The gestural and language skills of children with and without a LD are presented in Table 1.

**Table 1:** Descriptive statistics of gestural and linguistic skills in TD children and children with LD at 24 months.

	**TD (*****n*** **=** **32)**		**LD (*****n*** **=** **10)**	
	***M (SD)***	***Md (IQR)***	***M (SD)***	***Md (IQR)***
Hand points	15.31 (13.72)	11.50 (14.75)	17.20 (12.07)	14.50 (15.75)
Index-finger points	10.94 (13.46)	5.50 (18.50)	1.60 (2.63)	0 (4.50)
Vocabulary size in German^a^	154.81 (124.33)	105.0 (206.0)	25.10 (17.39)	26.0 (30.0)
Vocabulary size in the other language^a^	137.42 (126.98)	107.0 (195.0)	22.00 (24.06)	13.5 (42.0)
Conceptual vocabulary size^a^	233.90 (115.07)	225.0 (157.0)	36.50 (23.25)	29.5 (50.0)
Word comprehension^b^	50.66 (7.89)	51.0 (10.0)	40.70 (9.14)	39.5 (16.0)
Sentence comprehension^b^	49.29 (9.56)	54.0 (13.0)	35.40 (7.59)	35.0 (15.0)
Word production^b^	43.61 (7.85)	43.0 (10.0)	31.78 (3.73)	33.0 (7.0)
Sentence production^b^	44.00 (7.00)	42.0 (8.0)	34.40 (4.62)	35.0 (9.0)

a *Number of spoken words, measured with the parent questionnaire FRAKIS (39)*.

b *Standard T-scores, measured with the language test SETK-2 (38)*.

#### Data Analysis

Apart from the variables *word comprehension* and *word production* none of the language measures were normally distributed as indicated by the Shapiro-Wilk-test. Therefore, we used the non-parametric Mann-Whitney-U test for group comparisons and report Median (*Md*) and interquartile range (*IQR*) for distributions. For better interpretation of the results we report Cohen's *d* as effect size.

For classification of children as either LD or TD at 24 months using their pointing behavior at 12 months as criterium, we calculated the relative improvement over chance [RIOC; (42)]. RIOC incorporates the base rate and the selection rate of a developmental disorder and a screening. The rationale behind is that screenings for developmental disorders with a low base rate which randomly identifies all children as TD would reach high scores in specificity and accuracy and would thus be invalid. As the RIOC responds much more sensible in this case it is considered a superior indicator for the prognostic validity of a screening tool (42).

## Results

At 12 months, all 42 infants produced pointing gestures. Twenty-seven infants pointed at least once with the index finger and were therefore classified as index-finger pointers (64%), while the remaining 15 solely used whole-hand points and no index-finger points and were consequently classified as hand pointers (36%). The vast majority of the index-finger pointers (89%) pointed more than once with the index finger within a range from 2 to 45 index-finger points. The index-finger pointers produced more pointing gestures in total (*Md* = 27.0, *IQR* = 20.0) compared to the hand pointers (*Md* = 12.0, *IQR* = 13.0, *U* = 76.0, *p* = 0.001, *d* = 1.193). Moreover, index-finger pointers combined their pointing gestures more often with a vocalization (proportionately to the number of pointing gestures; *Md* = 0.47, *IQR* = 0.41) than hand pointers (*Md* = 0.24, *IQR* = 0.25, *U* = 107.5, *p* = 0.013, *d* = 0.834). The number of index-finger points produced by the caregivers of index-finger pointers (*Md* = 14.0, *IQR* = 18.0) and hand pointers (*Md* = 18.0, *IQR* = 25.0) did not differ (*U* = 201.0, *p* = 0.969, *d* = 0.012).

Comparing the language development of infants who were able to point with the extended index finger at 12 months to those infants who did not use the index finger for pointing at this young age resulted in differences between the two groups. Index-finger pointers showed an advanced language status at 24 months compared to hand pointers; they had a greater conceptional vocabulary and a better sentence comprehension (Table 2). Moreover, most of the index-finger pointers were typically developed at 24 months (89%) while nearly half of the hand pointers (47%) were language delayed. Table 3 summarizes the results of classifying index-finger pointers and hand pointers as either language delayed or typically developed. In other words, using the criterion of index-finger pointing at 12 months for identifying children with LD at 24 months reflects a sensitivity of 70%, a specificity of 75%, an accuracy of 74%, and a RIOC of 53% (see Table 4 for all commonly used values of quality criteria for screening tools).

**Table 2:** Comparison of language skills at 24 months between index-finger pointers and hand pointers.

	**Index-finger pointers**		**Hand pointers**				
	***Md***	***IQR***	***Md***	***IQR***	***U***	***p***	***d***
Conceptional vocabulary	225.0	199.0	105.0	132.0	102.0	0.12	0.891
Word comprehension	48.0	13.0	48.0	13.0	169.0	0.371	0.274
Sentence comprehension	54.0	14.0	41.0	13.0	114.0	0.026	0.768
Word production	39.5	12.0	37.0	15.0	136.5	0.196	0.555
Sentence production^*^	41.5	8.0	38.5	11.0	25.0	0.052	0.859

* *Since this subtest was only done with those children who were able to do it solely in German (n = 24) the group of index-finger pointers consisted of 18 children and the hand-pointers of 6*.

**Table 3:** Classification of children as being language delayed at 24 months based on their ability to produce index-finger points at 12 months.

		**LD at 24 months**		
		**Yes**	**No**	**Total**
Index finger pointing at 12 months	No	7 (70%)	8 (25%)	15 (36%)
	Yes	3 (30%)	24 (75%)	27 (64%)
Total		10 (100%)	32 (100%)	42 (100%)

${\text{χ}}_{\left(1\right)}^{2}$ *= 6.72, p = 0.010, Cramer's V = 0.40*.

**Table 4:** Quality criteria of index-finger pointing at 12 months as screening tool for LD at 24 months.

**Criterion**	**Value**
Sensitivity	0.70
Specificity	0.75
Positive predictive value	0.47
Negative predictive value	0.89
Accuracy	0.74
Selection rate	0.58
Relative improvement over chance (RIOC)	0.53

When directly comparing the results of the bilingual sample to those of a monolingual sample (5), there were no statistical significant differences in the number of index-finger pointers [$\chi_{\left(1\right)}^{2}$ = 1.72, *p* = 0.189, *Cramer's V* = 0.131] or the number of index-finger points produced (*Md*_*mono*_ = 6.0, *IQR*_*mono*_ = 16.0, *Md*_*bi*_ = 4.0, *IQR*_*bi*_ = 15.0, *U* = 1,071.0, *p* = 0.241, *d* = 0.232), although the effect sizes indicate small effects. There were no differences found in any gestural or language measures between children growing up with two or more languages.

## Discussion

The current study reproduced the pattern of earlier findings from a monolingual sample (5) on the relation between early pointing and language development and extended it to bilingual infants. Current main findings were that the absence of index-finger pointing at 12 months in a bilingual sample, like in a monolingual sample, indicates a higher risk for being language delayed at 24 months. These findings are in line with the current state of research showing that index-finger pointing reflects advances in children's communication, which leads to an earlier achievement of linguistic competencies (1, 4, 8). Bilingual 12-month-old infants in the current study who used index-finger points to communicate with their caregivers demonstrated better language skills at 24 months than infants who did not use index-finger points at 12 months. Based on the finding that just very few index-finger pointers were identified as having a LD at 24 months, while nearly half of the hand pointers had a LD at 24 months, it seems appropriate to consider index-finger pointing as a sign of TD and its absence as a risk factor for language acquisition.

In line with other studies reporting no differences in gestural development based on different cultural backgrounds or bilingualism (25–27, 29), we found neither differences in the number of index-finger pointers and hand pointers nor in the number of index-finger points produced between our presented bilingual sample and a monolingual sample using the identical procedures (5). Nevertheless, the screening criteria, specificity and RIOC of index-finger pointing, seem less robust in this bilingual sample compared to the monolingual sample (5). In the current bilingual sample 36% of the infants were classified as hand pointers while only 20% of the monolingual infants, investigated by Lüke et al. (5), did not produce index-finger points at 12 months. Possibly, this could be the result of the time point of data collection in some infants. In the monolingual sample eight infants could only be tested comparably late (16–37 days after their first birthday). Two of these slightly older infants did not point with the index finger and were later identified as being language delayed while the other six infants produced index-finger points and were not language delayed at 24 months. In the bilingual sample, a reverse pattern had occurred: The three bilingual infants who had been tested comparably late (23–30 days after their first birthday) did produce index-finger points and were identified as having a LD at 24 months. It remains unknown whether the results would have been identical if these infants had been observed 1 or 2 weeks earlier. These observations demonstrate the highly dynamic developmental pathways at this young age which may have affected the findings. Since the first productions of index-finger points occur between 10 and 12 months (43), further research with infants between 9 and 12 months of age is needed, so that the onset of index-finger pointing as predictor of later language skills can be analyzed and might be more robust as diagnostic tool compared to the absence of index-finger pointing at 12 months. These slightly differences reveal very clearly that the development of early communicative gestures is occurring at a rapid pace, resulting in a sudden change in categorization of a child as index-finger vs. hand pointer from 1 day to the next.

Beyond that, the sample sizes of both samples, the monolingual as well as the bilingual, are with 59 or 42 too small and not appropriate to prove any ability or tool as a prognostic valid screening instrument. The presented values of quality criteria for screening tools in this study as well as in the study with the monolingual sample (5) can only serve as orientation. Nevertheless, these orientating values with, for example good accuracies between 74 and 85%, the predictive value of pointing gestures found in many studies [for meta-analysis see (1, 6)], and the language and cultural universal occurrence of index-finger pointing (25–27, 29) are encouraging to further investigate the use of index-finger pointing as an early indicator of LD in a population-based study with children between 10 and 12 months during pediatric service. This would be especially important for children growing up with two or more languages since the identification of bilingual children with LD or even DLD is particularly challenging (23, 24).

## Data Availability Statement

The raw data supporting the conclusions of this article will be made available on request to the corresponding author.

## Ethics Statement

The study was reviewed and approved by the Internal Review Board of Bielefeld University (EUB 2015-079). Written informed consent to participate in this study was provided by the participants' legal guardians.

## Author Contributions

CL was responsible for data collection, data analysis, and drafted the first manuscript. All authors designed the study, developed the coding system, interpreted the data, and revised the manuscript critically.

## Funding

This project was funded by the Deutsche Forschungsgemeinschaft, LI 1989/2-2 to UL, LU 2116/1-2 to CL, and RI 898/6-2 to UR. This publication was supported by the Open Access Publication Fund of the University of Wuerzburg.

## Conflict of Interest

The authors declare that the research was conducted in the absence of any commercial or financial relationships that could be construed as a potential conflict of interest.

## Publisher's Note

All claims expressed in this article are solely those of the authors and do not necessarily represent those of their affiliated organizations, or those of the publisher, the editors and the reviewers. Any product that may be evaluated in this article, or claim that may be made by its manufacturer, is not guaranteed or endorsed by the publisher.
